# Pre-Service Teachers’ Intervention in School Bullying Episodes with Special Education Needs Students: A Research in Italian and Greek Samples

**DOI:** 10.3390/ijerph15091908

**Published:** 2018-09-02

**Authors:** Tatiana Begotti, Maurizio Tirassa, Daniela Acquadro Maran

**Affiliations:** 1 Department of Psychology, Università di Torino, Via Verdi 10–10124 Torino I, Italy; tatiana.begotti@unito.it (T.B.); maurizio.tirassa@unito.it (M.T.); 2 Center of Cognitive Science, Università di Torino, Via Po 14–10123 Torino I, Italy

**Keywords:** bullying, intervention, pre-service teachers, special education needs students

## Abstract

*Background*: The aim of the study was to compare the level of self-confidence in dealing with problems at school, the attitude towards bullying situations and the recommended strategies to cope with bullying in two samples of pre-service teachers (PSTs). The PSTs were in training to become teachers with special education needs students (SEN) and came from two different countries (Italy and Greece). *Methods*: A questionnaire survey was made involving 110 Italian and 84 Greek PSTs. *Results*: The results about self-confidence showed that Greek PSTs had lower outcome expectations and a higher external locus of causality than Italian PSTs. Teachers’ training programs and school preventive intervention were also discussed. *Conclusions*: Because the participants in this investigation will be teachers in the near future, they require specific training on bullying in general and in students with SEN in particular.

## 1. Introduction

Bullying is a well-known problem that involves students in primary and secondary school. The phenomenon is defined as an aggressive behaviour, repeated over time, in which the victim perceives a power imbalance [1,2]. The different forms of bullying are classified on the basis of the overt/covert dichotomy: overt bullying includes physical aggressions such as hitting, punching or kicking, or verbal aggressions such as insults or threats; covert bullying is a type of relational aggression and includes less visible actions such as gossiping, social exclusion or isolation [3,4]. Early research addressed students with Special Education Needs (SEN) and bullying victimization. Rose and Cage [5] showed students with disabilities and/or SEN to be generally more involved in the dynamics of bullying; they also turn out to be engaged in higher levels of perpetration than their peers without disabilities. Similarly, in their investigation about bullying among students with and without SEN, Fink et al. [6] argued that children with SEN are at greater risk of victimization (covert and overt) than other students. While children with SEN have a victimization rate between 30% (children with reading difficulties [7]) and 83% (children with learning difficulties [8]), children without SEN have a victimization rate of <20% [9]. Thus, SEN increases vulnerability to bullying and bullying in its turn increases distress and may inhibit the child’s capability of entertaining positive social interactions in school and in other contexts [1]. McLaughlin and colleagues [10] suggested that this vulnerability was related to peer isolation, relational difficulties and poor acceptance in classrooms. In detail, the motives of the victimization are well explained by Pavri and Luftig [11]. The authors suggest that students with SEN demonstrate poor social competence: for example, delays in social development and lack of skills in initiating and sustaining positive social relationships and interpreting social cues. Moreover, they often demonstrate pervasive deficits in social functioning, exhibiting more aggressive and negative behaviours. In many ways, students with SEN have less sophisticated social skills that lead them to misinterpret social cues and to use ineffective responses [12]. Consequently, peers use covert behaviour, such as rejecting or ignoring them, instilling a sense of loneliness in students with SEN [13]. As underlined by Bryant, Smith and Bryant [14], inclusion is considered the best way to respond to the needs of students with SEN to avoid margination: inclusive education has a positive effect on social functioning [15,16,17]. To build an inclusive classroom and to take care of students (to respond to their emotional and psychosocial needs), teachers undoubtedly play a central role [18,19,20]: in the classroom, the teacher could create a context to enable students with and without SEN to learn and develop social competence, learning from each other. Moreover, because the highest victimization rate is in students with SEN, the teacher should pay particular attention to bullying [21]. Nevertheless, an analysis of the literature indicates that teachers tend to underestimate bullying episodes (particularly covert bullying) when reported by students with SEN, thus ignoring the victimization [10]. At the same time, research has shown that teachers play a key role in intervening to stop it, for example, by managing the classroom and/or suggesting ad hoc coping strategies to the victim [22,23]: fostering social development in the classroom may yield more appropriate interactions in social settings, also allowing bystanders and other third parties to intervene more often and/or more strongly in the defence of the victim.

Coping Strategies. Davis and Nixon [9] conducted an interesting study to evaluate students’ perceptions about the effectiveness of the strategies used to reduce both overt and covert bullying at school. Their findings confirmed that students with SEN reported higher levels of victimization due to their disability. Regarding strategies to cope with the phenomenon, about one-third of students suggested that accessing support from peers and adults was the most helpful strategy. In particular, students appreciated adults telling them that they did the right thing to report what was happening, suggesting conflict resolution and/or the use of assertive communication. These adults listened and encouraged students, telling them that bullying was not their fault and suggesting that things would get better. However, the interventions of more than one-third of adults at school were described by victims as inefficient and did not affect their victimization. Some students reported that the intervention of one-third of adults at school made things get worse. The adults may have blamed the student for the victimization, told them to stop reporting it to adults, or instructed them to tell the bully how the victim feels (e.g., crying, venting), solve the problem him/herself (e.g., planning for revenge) and not tattle (e.g., ignoring). Scolding the victim for tattling was reported by the students as having the most negative impact in stopping the bully. Nevertheless, the authors stated that students with SEN reported that adults told them not to tattle almost twice as often as students without SEN. As emphasized by Cortes and Kochenderfer-Ladd [24], however, not only are avoidance behaviours generally ineffective but they may even have a reverse buffering effect that worsens the situation. If the teacher fails to express concern or to share appropriate advice about bullying, the students may feel uncomfortable approaching him/her not only as far as bullying is involved but also for guidance and support for other problems. On the other hand, Sokol et al. [25] examined the teachers’ perspectives in responding to overt and covert bullying. Their study aimed to investigate the strategy perceived to be most effective. Their findings showed that participants were most likely to suggest to victims to report the episode to school staff. At the same time, teachers indicated that suggestions were related both to circumstances and the victim’s characteristics: in some cases, the teacher recommended a contradictory approach, suggesting that they ignore the bully and express their feelings (venting). In other cases, teachers’ recommendations were vague or insufficient to help the victims to stop the bullying. Thus, teachers may give conflicting and confusing messages to victims that could be perceived as useless. Therefore, because students will ask for help from adults, teachers must be ready to intervene in bullying episodes, suggesting more effective coping strategies. In particular, when students involved in bullying episodes have SEN [21], a strategy that worsens the situation could isolate them even more. The teacher’s ability to intervene effectively in bullying cases was associated with the confidence that she/he has the capability to address problems at school [25,26,27]. As suggested by Roland and Galloway [28], students are less likely to become involved in bullying episodes when they perceive that teachers pay attention to them, particularly when a teacher is able to care for students, promote a positive environment in the classroom [29,30] and manage matters of learning and behaviour in a positive way. In the literature, the feeling of confidence was related to several explanations [25,31]. Among them, authors such Denzine, Cooney and McKenzie [32] described self-efficacy, outcome expectations and causal explanations given to each episode of bullying. According to Bandura [33], self-efficacy is the individual’s perception of being able to cope with a certain task. In teachers, this perception is about his/her capability to produce outcomes (e.g., student engagement and learning) even if students are unmotivated or difficult [34]: teachers who sense a good relationship with their classroom tend to express higher level of self-efficacy. Self-efficacy is related to the chance of successfully intervening with bullying episodes. Findings from an investigation by Veenstra et al. [35] showed that classes in which teachers had a high self-efficacy also had lower occurrences of bullying. Thus, if teachers consider a bullying episode as serious, they are more likely to intervene if they have the knowledge and skills to act effectively [36,37]. A related notion is outcome expectations; that is, the belief that the behaviour will follow as a consequence of a specific outcome [38]. Teachers’ expectation about students’ outcomes affect the behaviour and the type and quality of interaction in the classroom [39]. If teachers have high expectations, they build a positive emotional environment [40] and increase their efforts to cope with problematic situations that occur in a classroom [41]. Social cognitivists make a distinction between outcome expectations and locus of causality [42]. The locus of causality has been described as the expectation of being able to control or reinforce the environment [43,44]. As underlined by Wang et al. [45], teachers tend to ascribe student’s failure to external causes. These could be related to social and family distress or personal problems. Conversely, students’ success is attributed to an internal cause, for example, the teachers’ ability to challenge the student and stimulate interest in the subject. The internal locus of causality permits the teacher to intervene actively in a problematic situation that occurs in the classroom [46], such as bullying episodes. Pre-Service Teachers [PST], that is, persons who have no teaching experience but are enrolled in a training programme (like a university or post-university course) preparing them to become in-service teachers [IST], are a particularly interesting population. Authors [47] found that PSTs are more likely than ISTs to display a low external locus of causality and to ascribe positive changes in the relationships between students to their own behaviour in the classroom. PSTs may also feel more secure about their capability of supporting the victims, for example, by encouraging proactivity on the part of the bystanders [27] but not in the families’ involvement. As suggested by Bagley, Woods and Woods [48], the parent’s involvement is particularly important when students with SEN are bullied. PSTs may also feel that they would more likely intervene in response to overt aggression than to covert [49]; however, as described above, students with SEN are more likely to be the targets of covert bullying. This may signal that these teachers had not (or not yet) been trained and sensitized to properly address bullying, particularly when pupils with SEN are involved [47,48]. In general, too high expectations on the part of PSTs may end up in burnout when unsupported by reality [50]. Specific training on bullying, its consequences in the life of the victims, the third parties and the bullies themselves and how to recognize and effectively cope with it, could then be useful both to enable the prospective teachers to handle the actual situations in which they will likely find themselves and to help prevent consequences on their mental health and professional well-being.

Current Study. Based on this literature review, we compared two samples of pre-service teacher (PST) training to become special education teachers from two different countries (Italy and Greece) characterized by different levels of problem severity. In particular, we intended to compare (i) the level of self-confidence in dealing with problems at school (self-efficacy beliefs, outcome expectations and locus of causality) of Italian and Greek PSTs; (ii) the attitude towards bullying situations (perceived seriousness of bullying, empathy with the victims and likelihood of intervention) of Italian and Greek PSTs; and (iii) the strategies of intervention in bullying situations recommended by Italian and Greek PSTs. (iv) We also intended to analyse the relationship between the recommended strategies to cope with bullying and the self-confidence and the attitude towards bullying in Greek and Italian PSTs. Previous research showed that PSTs perceive themselves as capable of effective intervention in bullying episodes (e.g., by encouraging viewers to be more pro-active) [27] when there was an overt episode [51]. However, Purdy and Mc Guckin [21] underlined that PSTs were not trained and sensitized to properly address the problem, particularly when the student involved in the bullying episode had SEN. In their investigation, the authors asked PSTs to describe their experience of dealing with bullying incidents (including incidents involving children with SEN) during school placement. The findings showed a lack of preparation of PSTs as a result of the lack of training on disablist bullying and “a subsequent lack of confidence in dealing with such incidents which were found to be often challenging and complex” [21] (p. 202). A European study on bullying found that in Italy, in a sample of 5042 students in secondary school, 15% declared themselves to be victims of bullying. Among 4987 students in Greece, this percentage was more than doubled (33%). The cause of bullying episodes was ascribed to a disability in 32% of cases in the Italian sample and 24% of cases in the Greek sample. Moreover, the findings showed that information about bullying (e.g., what this phenomenon is, how to cope with a bully, etc.) came mainly from school in both countries (30% in Italy, 39% in Greece). School is considered the most suitable place to talk about bullying (30% and 26%, respectively) and the most common coping strategy for victims was to ask for help from teachers (respectively 29% and 30%) [52]. Based on the above data, the aim of this study was explorative: the novelty of this work lies in the fact that, for the first time, self-confidence, attitude towards bullying and suggested strategies were compared in two PST samples of different nationalities. In particular, bullying is more common in Greece than in Italy and a greater proportion of people with disabilities are victims in Italy than in Greece.

Our expectations were the following:

1. We expected both Italian and Greek PSTs to have: (i) high levels of self-confidence in their capability of coping with problems at school, (ii) high levels of self-efficacy and expectations of outcomes and (iii) a prevalent internal locus of causality [47].
2. We (i) expected both Italian and Greek PSTs to perceive bullying, especially when overt, as a serious problem and to feel empathy toward the victims; however, (ii) we expected a higher likelihood of will to intervene in the Italian sample, due to the higher degree of victimization in Greece [52].
3. We expected in both the Italian and the Greek PSTs: (i) the strategies “Tell someone” and “Tell the bully how the victim feels” to be positively related to self-confidence, perceived seriousness of the episode, empathy with the victims and likelihood of intervention and (ii) the strategies “Solve the problem himself” and “Do not tattle” to be negatively related to the same variables [25].

To understand the perception that PSTs have both of bullying and of their own means to cope with it would allow to better tailor courses aimed at improving their relevant skills. Both the investigation and the intervention are particularly needed in countries where bullying appears to be more widespread and even more so where students with disabilities are particularly targeted.

## 2. Method

### 2.1. Ethical Statement

The study presented in this article conformed to the provisions of the Declaration of Helsinki in 1995, revised in Edinburgh 2000 [53]. All ethical guidelines were followed, as required for conducting human research, including adherence to the legal requirements of Italy. The research project was approved by the Directors of the Master. Since there was no medical treatment or other procedures that could cause psychological or social harm to participants, additional ethical approval was not required. With the approval of the Directors of the Master, participants were asked for authorization to administer the questionnaire. The cover sheet clearly explained the research aim, the voluntary nature of participation, the anonymity of the data and the elaboration of the findings. Thus, returning the questionnaires implied consent. Participants volunteered in the research without receiving any reward.

### 2.2. Participants

A total of 194 PSTs aged 22–52 (mean age 28 years, SD = 6.94) provided the data. The majority of respondents were female (179; 92%), confirming the trend in most countries [54,55]. Approximately 110 (57%) PSTs were Italian, aged 22–52 (mean age 27 years, SD = 6.8), with 96% females. They were recruited from the University of Torino (Italy), where they were attending the last year of the Master’s course in Education. Eighty-four (43%) PSTs were Greek, aged 23–51 (mean age 29 years, SD = 6.94), with 87% females. They were recruited from the University of Torino, where they were attending a Master’s course in Relational Competence in Teaching. The Greek students had courses in English, so they had to verify their knowledge of the language upon registration. Both groups (Italian and Greek students) were attending a course in preparing teachers to teach students with SEN. In most cases, Greek PSTs had work experience in teaching (68; 82.9%): for 38 PSTs, the duration of teaching experience was less than five years and for the rest of the sample, it was more than five years. Italian PSTs had teaching experience in 52 cases (47.3%): for 31 PSTs, the duration was less than five years and for 21, it was more than five years (χ^2^ = 25.65; *p* = 0.000).

### 2.3. Materials

Participants were asked to anonymously fill out a self-administered questionnaire consisting of several sections. The first section described the purpose of the investigation and contained the instructions for replying, the anonymity and privacy statements and questions about the respondent’s sex and age. The second part aimed to investigate the participants’ self-confidence in dealing with problems at school, their attitudes towards bullying and victimisation in the case of overt and covert bullying and the strategies suggested to stop bullying. The measures used in this study were the Teacher Efficacy Scale (TES) [56], the Bullying Attitude Questionnaire [57] and the recommended strategies for coping with bullying. We used the three-factor model of TES [32], which included (i) three items measuring self-efficacy beliefs (SEB) (e.g., “If one of my students couldn’t do a class assignment, I would be able to accurately assess whether the assignment was at the correct level of difficulty”); (ii) three items measuring outcome expectation (OE) (e.g., “If a student masters a new concept quickly, this might be because I knew the necessary steps in teaching that concept”); and (iii) four items measuring external locus of causality beliefs (E-LOC) (e.g., “A teacher is very limited in what he/she can achieve because of a student’s home environment”). Each item was measured on a five-point scale (from 1 = strong disagreement, to 5 = strong agreement). Reliability, assessed with Cronbach’s alpha, was SEB α = 0.54 (range 3–15); OE α = 0.63 (3–15); E-LOC α = 0.53 (4–20).

The Bullying Attitude Questionnaire included six scenarios hinging on either overt (such as physical or verbal) or covert (such as social exclusion) bullying episodes. Following the description of the scenarios, three items sought to evaluate the perceived seriousness of the episode (measured on a five-point scale from 1 = not serious, to 5 = very serious), the degree of empathy towards the victim (measured on a five-point scale from 1 = strong disagreement, to 5 = strong agreement) and the likelihood of intervention (measured on a five-point scale from 1 = not likely, to 5 = very likely). The six subscales were thus aimed at evaluating the three dimensions described above (perceived seriousness of the episode, empathy towards the victim and likelihood of intervention) in overt and covert bullying, each represented in three scenarios. Each subscale ranged between 3–15 points. The reliability of the subscales was measured with Cronbach’s alpha: seriousness of overt bullying (α = 0.65); seriousness of covert bullying (α = 0.64); empathy in overt bullying (α = 0.82); empathy in covert bullying (α = 0.74); likelihood of intervention in overt bullying (α = 0.66); and likelihood of intervention in covert bullying (α = 0.60).

Regarding the recommended strategies for coping with bullying, participants were asked which strategies they would recommend to a child victim of bullying. Then, they were asked about their usage of ten different strategies, responding yes (score 2), sometimes (scored 1) or no (scored 0). In line with previous research [9], we divided the strategies in four categories: (1) accessing support from peers and adults (3 items, ranged 0–6; for example, “Tell parents”); (2) telling the bully how the victim feels (2 items, ranged 0–4; for example, “Crying”); (3) solving the problem him/herself (3 items, ranged 0–6; for example, “Fighting”); and (4) instructing them not to tattle (2 items, ranged 0–4; for example, “Ignoring”) (Cronbach’s alpha α = 0.53).

For the Italian sample, we used the Italian version of the questionnaire, which was already used in an investigation on bullying involving teachers [21]. Statistical analyses were performed with SPSS, version 22 (SPSS Inc., Chicago, IL, USA). Descriptive measures (means ± SD) were calculated for all test variables for all groups of participants. The different scores were examined using analysis of variance (ANOVA); eta squared was calculated to estimate the effect size. Correlations and regression analysis were calculated to examine the relationship between self-efficacy beliefs, outcome expectations, locus of causality, perceived seriousness of bullying, empathy with the victims, likelihood of intervention and strategies recommended to cope with bullying, in each group of participants (Italian and Greek PSTs).

### 2.4. Procedure

The data were collected by one of the authors of this paper and by assistants trained by the researchers. The participants were contacted through their academic courses and were informed that they were participating in a study to investigate the bullying phenomenon in their own point of view. Data collection involved completion of a structured questionnaire submitted on paper. All the participants were informed that participation was voluntary and that their responses were anonymous. The self-report questionnaire (both the Italian version and the English version) took approximately 20 min to complete. The participants were asked to insert the completed questionnaire in a slot box, so to guarantee anonymity. All the questionnaires were group administered in classrooms in a single day, with the permission of the Directors of the Master before the beginning of a lesson and were returned immediately. The response rate in both groups was 100% and all questionnaires were completely filled in. Therefore, none was excluded. The study was conducted in accordance with privacy requirements. This procedure was in accordance with the code of ethics of the Italian Association of Professional Psychologists and Italian law (the latter concerning privacy).

## 3. Results

The first aim of this study was to compare the level of self-confidence in dealing with problems at school (distinguishing between self-efficacy, outcome expectations and locus of causality) of Italian and Greek PSTs. The results are reported in Table 1.

The second aim of this study was to compare the attitude towards bullying situations (distinguishing between perceived seriousness of bullying, empathy with the victims and likelihood of intervention) in Italian and Greek PSTs. The results are reported in Table 2.

As shown in Table 2, no statistically significant differences were found between Italian and Greek PSTs concerning perceived seriousness of bullying and the likelihood of intervention in cases of both overt and covert bullying. Furthermore, Greek PSTs reported a lower level of empathy than Italian PSTs in cases of both overt and covert bullying.

The third aim was to compare the strategies of intervention in bullying situations recommended to students by Italian and Greek PSTs. The results are reported in Table 3.

As shown in Table 3, Greek PSTs suggested the strategies “Tell someone” and “Solve the problem himself” less than Italian PSTs, while they suggested the strategy “Do not tattle” significantly more than Italian PSTs. No difference was found in the strategy “Tell the bully how the victim feels.”

The fourth aim was to analyse the relationship between recommended strategies to cope with bullying and self-confidence and the attitude towards bullying in Greek and Italian PSTs. The results are reported in Table 4 and Table 5.

As shown in Table 4, in Greek PSTs, we found a significant and positive correlation between the strategy “Solve the problem himself” and an external locus of causality. Regression analysis was performed, using “external locus of causality” as independent variable and “solve the problem himself” as dependent. Results shown a significant causal relation between variables (β = 0.35; *p* = 0.032; R^2^ = 0.13).

As shown in Table 5, in Italian PSTs, we found significant and positive correlations between the strategy “Tell someone” and empathy and likelihood of intervention in cases of both overt and covert bullying. Regression analysis was performed, using “empathy (covert)”as independent variable and “tell someone” as dependent. Results did not show significant causal relation between variables (β = 0.13; *p* = n.s.; R^2^ = 0.017). Also, the regression analysis performed with “intervention (covert)” as independent variable and “tell someone” as dependent did not show significant causal relation between variables (β = 0.10; *p* = n.s.; R^2^ = 0.010).

## 4. Discussion

This study aimed to investigate the differences between Italian and Greek PSTs (students in training to become special education teachers) concerning the strategies of intervention against bullying at school. According to the literature, the ability to cope with bullying is associated with self-efficacy, outcome expectations and the causal explanation of the phenomenon. The first hypothesis of this study was partially confirmed: the results showed that there are no differences between Italian and Greek PSTs in self-efficacy: one of the main sources of self-efficacy is the direct experience of success. An explanation could be in the characteristics of the participants: because they were PSTs, they did not have direct classroom management experience (except for traineeships). However, Italians and Greeks differed in their expectations of success (higher in Italians) and the external locus of causality (higher in Greeks). This appears to imply that the Italians had a higher expectation of success in managing the class. As pointed out above, Gold & Roth [50] suggested that high expectations unsupported by reality can end up in burnout. This is likely to affect the Italian PSTs in particular. On the other hand, Greek PSTs showed a greater external locus of causality (that is a stronger tendency to attribute causal responsibility to others than to oneself): thus, they were more at risk of underestimating the importance of their role in managing the classroom, shifting responsibility to the families and/or the social context. This could also hamper the attempts to create a positive context, as free as possible from aggressive covert and overt behaviour towards students with and without SEN [18,23].

The second hypothesis of this study was partially confirmed: the findings showed that Italians and Greeks PSTs perceived bullying as a serious problem and had a high propensity for intervening, both in overt and covert episodes. This is an interesting finding because, as suggested by Zee and Koomen [36], if teachers consider a bullying episode as serious, they are more likely to consider their intervention as essential [38]. Moreover, these results are not in line with Kahn and colleagues [49] because we did not find a greater attitude to intervene in the case of overt bullying. There is, however, a difference in the case of empathy: Greek PSTs have a lower level of empathy towards the victim in both overt and covert bullying. This finding is particularly interesting: from the literature, empathy is an important predictor of intervention [3]. Finally, the lack of empathy towards the bullied pupils might influence the students’ confidence in approaching the teacher about other types of problems, leading them to renounce an important guide. This is particularly true for students with SEN, who need support and information to resolve problems and difficulties [24].

Regarding strategies, the results showed a significant difference among Italian and Greek PSTs. Italian PSTs more often suggest the ‘Tell someone’ and ‘Solve the problem him/herself’ strategies, while Greek PSTs more often suggest the ‘Do not tattle’ strategy. An interesting finding concerns the expression of the emotion: the ‘Tell the bully how the victim feels’ strategy was not significantly different among Italian and Greek PSTs. Davis and Nixon [9] showed that students victims of bullying found the ‘Tell someone’ strategy useful, while ‘do not tattle’ was less effective. Thus, the suggestion of the strategy ‘Do not tattle,’ especially used with students with SEN, means that the victim of bullying does not feel helped, risking further isolation and a lack of understanding. In accordance with Rigby [58], the result is that victims of bullying risk experiencing frustration in the need for help.

Coming to the third hypothesis, only in few cases relations were found between self-confidence, perceived seriousness of bullying, empathy with the victim, proneness to intervention and the different strategies. In particular, the strategy ‘solve the problem him/herself,’ while recommended by Italian PSTs, was only related to a high external locus of causality in Greek PSTs. Regression analysis confirmed a causal relation between these two variables. This means that Greek PSTs are more likely to suggest to the victims to find a solution themselves, indicating the PST’s inability to intervene effectively when they attribute an external locus of causality to the problem into the classroom. Because an external locus of causality attributes the failure to social and family distress or personal problems [45], PSTs probably suggest this coping strategy to attribute the cause (and thus the solution) to the victim.

As described above, Italian PSTs are more prone to suggesting ‘Tell someone’ or ‘Solve the problem him/herself’ than Greek PSTs. As previously mentioned, victims found that ‘Tell someone’ was particularly useful [9], unlike ‘Solve the problem him/herself.’ The strategy ‘Tell someone’ could be counterproductive (reverse buffering effect) [59] if the person asked for help, such as the teacher, suggests a poor strategy. This strategy was more commonly suggested by Italian PSTs with higher scores in empathy and a higher propensity to intervene in covert bullying episodes. This finding appears to be very interesting despite regression analysis did not confirm the causal relation between the variables. According to the literature, teachers tend to underestimate covert bullying [60,61] and are more likely to intervene in the cases of physical and verbal bullying and to take no action in cases of social bullying [62]. Thus, this finding is not in accordance with the literature, since Italian PSTs suggest that covert bullying victims talk about their experience. This strategy allows victims to escape the sense of isolation that characterizes bullying victims in general and the covert bullying victims in particular.

There are, of course, limitations to this study. First, since participants belonged to a sample of convenience, non-randomly selected, the results should be considered with caution and not be generalized. Moreover, the participants’ possible previous experience with bullying (as a victim, bully, bystander, or other role) was not investigated: such experience, however, would likely affect one’s perception [63]. Third, the questionnaire used in this research sought to investigate the PST’s attitudes towards bullying in the classroom and not towards bullying in students with SEN. Future research should be addressed to investigate previous experience in bullying in PSTs and their attitude towards bullying in students with SEN. Finally, the questionnaire did not take into account the PST’s confidence in dealing with cyberbullying. This, too, should be the matter of future investigations.

## 5. Conclusions

Despite these limitations, this investigation suggests particular strategies for Italian and Greek PST training. Because the participants in this investigation will be teachers in the near future, they require specific training on bullying in general and on bullying in students with SEN in particular. Concerning the former, training might include discussions of overt and covert bullying aimed at helping distinguish it from other forms of aggression (not repetitive, not imbalanced and/or not intentional). This could also include examples of intervention, both toward the bullies and toward the victims. Baumann and Del Rio [64] suggested to use videos, role play and other available techniques aimed at the understanding of theories and at the application of recommended strategies of intervention in simulated situations. Training could also improve the ability of promoting empathy and pro-social skills not only in bullies and victims but in the entire classroom. Furthermore, training should highlight the central role that teachers can have in stopping the phenomenon. In fact, suggesting a strategy and accepting the victim’s need for help can disrupt the victim-bully relationship and influence children’s attitudes on bullying and bystanding behaviour [26,65]. Specifically, students with SEN should be helped effectively and in a timely manner. These students are particularly vulnerable and need to be placed in a class that can accommodate them, not isolate them. Inclusive education itself may offer opportunities for students with SEN to interact with peers but it does not necessarily lead to positive and supportive relationships [66]. This suggestion is specific for Italian PSTs, where the bullying affects students with SEN more than in Greece. Moreover, for Italian PSTs the courses ought to take into account the issue of the high outcome expectations, so to avoid the risk, discussed above [50], that these teachers develop unrealistic expectations, unsupported by reality. Furthermore, as suggested by Bagley, Woods and Woods [48], training courses ought to stress the importance of parents’ involvement in cases of bullying, especially where students with SEN are involved. This is important for all PSTs, particularly for the Greek ones, whose high external locus of causality could lead to handing over the whole responsibility of intervention to the families. This would have several negative consequences, among which the failure to build an effective alliance with them. As shown by Nicolaides, Toda and Smith [26], specific training in university courses could improve teachers’ chances of a more effective intervention. These courses should focus not only on the mere concept of bullying, its nature and causes, the protagonists and the possible coping strategies with respect to the perpetrators, the victims, the classroom and others but also on the specific case when students with SEN are involved. If PSTs have expertise on the issue, they could be better able to manage and intervene effectively against bullying. This could disrupt the chain of victimization, benefiting not only the victims and the perpetrators but also the classroom as a whole and the teacher’s well-being.

## Figures and Tables

**Table 1:** Self Confidence in dealing with problems at school. Comparison between Italian and Greek PST (one-way ANOVA).

	Italian PST (n = 110)	Greek PST (n = 84)			
	*M (SD)*	*M (SD)*	*F*	*p*	*η^2^*
Self-efficacy beliefs (range 3–15)	9.20 (1.573)	9.17 (2.159)	0.020	n.s.	0.000
Outcome expectations (range 3–15)	10.28 (1.848)	8.90 (2.234)	14.437	0.000	0.089
External locus of causality (range 4–20)	9.99 (2.179)	11.74 (2.414)	17.542	0.000	0.107

Note. *M* = mean; *SD* = standard deviation; *F* = Fisher’s ratio; *p* = *p* value; n.s. = not statistically significant; *η*^2^ = eta square; As shown in Table 1, Greek PSTs had lower outcome expectations and a higher external locus of causality than Italian PSTs. No differences were found in self-efficacy beliefs.

**Table 2:** Attitude toward bullying. Comparison between Italian and Greek PST (one-way ANOVA) (Range: 3–15).

	Italian PST (n = 110)	Greek PST (n = 84)			
	*M (SD)*	*M (SD)*	*F*	*p*	*η* ^2^
Seriousness (overt)	12.63 (1.842)	12.64 (1.857)	0.002	n.s.	0.000
Seriousness (covert)	12.78 (1.829)	12.82 (1.904)	0.013	n.s.	0.000
Empathy (overt)	11.71 (2.421)	10.79 (3.223)	3.409	0.048	0.151
Empathy (covert)	11.61 (2.333)	10.58 (2.992)	4.683	0.032	0.177
Intervention (overt)	12.84 (1.904)	12.67 (2.442)	0.196	n.s.	0.001
Intervention (overt)	12.82 (1.897)	12.46 (2.234)	0.925	n.s.	0.006

Note. *M* = mean; *SD* = standard deviation; *F* = Fisher’s ratio; *p* = *p* value; n.s. = not statistically significant; *η*^2^ = eta square.

**Table 3:** Strategies of intervention. Comparison between Italian and Greek PST (one-way ANOVA).

	Italian PST (n = 110)	Greek PST (n = 84)			
	*M (SD)*	*M (SD)*	*F*	*p*	*η* ^2^
Tell someone (range 0–6)	5.66 (0.67)	5.21 (1.24)	8.14	0.005	0.053
Tell the bully how the victim feels (range 0–4)	1.91 (1.97)	1.84 (0.99)	0.14	n.s.	0.001
Solve the problem himself (range 0–6)	2.16 (0.97)	1.68 (0.88)	7.10	0.009	0.048
Do not tattle (range 0–4)	1.46 (1.33)	2.56 (1.14)	20.98	0.000	0.128

Note. *M* = mean; *SD* = standard deviation; *F* = Fisher’s ratio; *p* = *p* value; n.s. = not statistically significant; *η*^2^ = eta square.

**Table 4:** Correlations among variables in Greek PST (N = 84).

	Tell Someone	Tell the Bully How the Victim Feels	Solve the Problem Himself	Do Not Tattle
Self-efficacy beliefs	0.12	−0.20	0.21	0.18
Outcome expectations	−0.06	0.24	0.19	0.16
External locus of causality	0.13	−0.16	0.35 *	0.03
Seriousness (overt)	0.18	0.19	0.18	−0.08
Seriousness (covert)	0.12	0.24	0.05	−0.16
Empathy (overt)	0.07	0.10	0.13	0.27
Empathy (covert)	0.13	0.04	0.16	0.22
Intervention (overt)	−0.06	0.04	−0.06	0.13
Intervention (covert)	0.10	−0.20	−0.24	0.03

Note: * *p* < 0.05.

**Table 5:** Correlations among variables in Italian PST (N = 110).

	Tell Someone	Tell the Bully How the Victim Feels	Solve the Problem Himself	Do Not Tattle
Self-efficacy beliefs	−0.13	−0.01	0.17	0.001
Outcome expectations	−0.12	0.01	0.09	0.14
External locus of causality	−0.09	0.12	0.07	0.02
Seriousness (overt)	0.07	0.05	−0.10	−0.08
Seriousness (covert)	0.16	−0.03	−0.15	0.09
Empathy (overt)	0.12	−0.04	−0.09	0.07
Empathy (covert)	0.25 **	0.04	−0.11	0.08
Intervention (overt)	0.17	0.17	−0.13	−0.08
Intervention (covert)	0.31 **	0.08	−0.10	−0.05

Note: ** *p* < 0.01.
